# Superoxide dismutase SOD-3 regulates redox homeostasis in the intestine

**DOI:** 10.17912/micropub.biology.001061

**Published:** 2023-12-22

**Authors:** Chiong-Hee Wong, Md Ahsanul Haque, Howard C. Chang

**Affiliations:** 1 Department of Emergency Medicine, Mackay Memorial Hospital, Taipei, Taiwan; 2 Cell Biology and Neuroscience, Rowan University School of Osteopathic Medicine

## Abstract

There are four cellular superoxide dismutase paralogs in *C. elegans* . The role of these superoxide dismutases in redox homeostasis remains largely unknown. Here, we generated the integrated redox reporter rxRFP to detect changes in redox homeostasis using live fluorescence imaging. We found that *sod-3* deletion contributes to oxidative stress elevation. Deletions in additional paralogs ameliorate the oxidative stress of *sod-3* . Complete elimination of all four paralogs elicits the same increase in rxRFP fluorescence as in the *sod-3* single mutation, suggesting a compensatory role of other *sod* s. Our results suggest that SOD-3 plays a key role in regulating gut redox homeostasis.

**Figure 1. SOD-3 plays a key role in regulating oxidative stress in the intestine:**
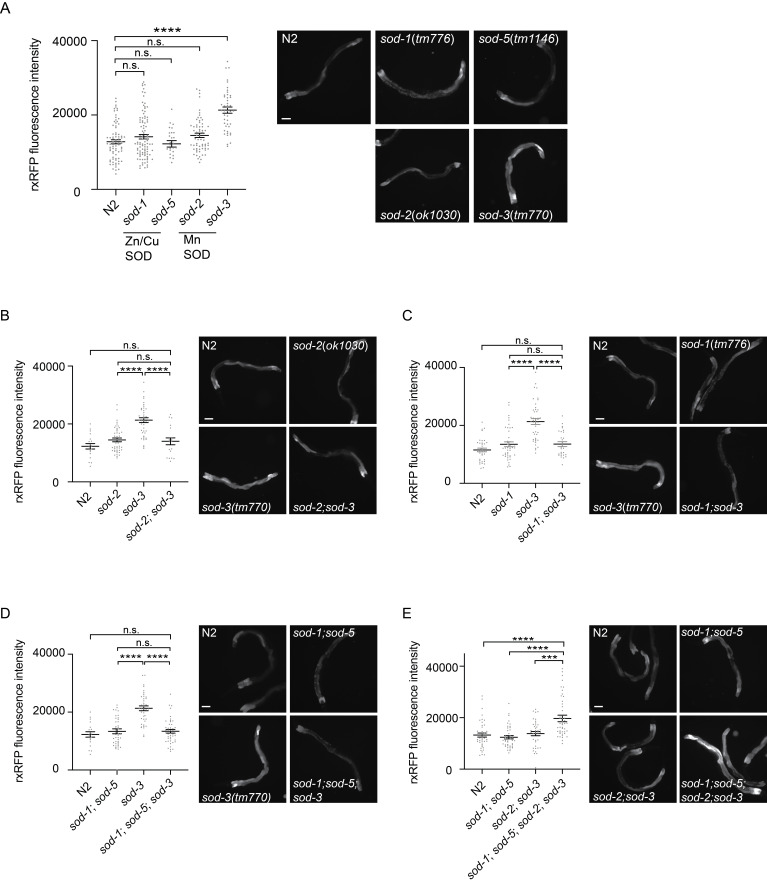
(A) Intestine-specific rxRFP fluorescence intensity is elevated in the *sod-3* ( *tm776* ) background. Left: fluorescence intensity of intestine-specific rxRFP in *sod-1* ( *tm776* ), *sod-5* ( *tm1146* ), *sod-2* ( *ok1030* ), and *sod-3* ( *tm770* ). Right: representative fluorescence micrographs. Error bar represents s.e.m., **** represents *p* < 0.0001, and ns represents not significant, as determined by one-way ANOVA, followed by Tukey’s multiple comparison analysis. (B) *sod-2* ( *ok1030* ) suppresses the *sod-3* ( *tm770* )-dependent rxRFP fluorescence increase. (C) *sod-1* ( *tm776* ) suppresses the *sod-3* ( *tm770* )-dependent rxRFP fluorescence increase. (D) The *sod-1* ( *tm776* ) and *sod-5* ( *tm1146* ) double mutant suppresses the *sod-3* ( *tm770* )-dependent rxRFP fluorescence increase. (E) The *sod-1* ( *tm776* ), *sod-5* ( *tm1146* ), *sod-2* ( *ok1030* ), and *sod-3* ( *tm770* ) quadruple mutant elicits enhanced gut rxRFP fluorescence. (B-E) Error bar represents s.e.m., **** represents *p* < 0.0001, *** represents *p* < 0.001, and ns represents not significant as determined by one-way ANOVA, followed by Tukey’s multiple comparison analysis. All worms were grown on *E. coli* OP50 at 20 °C prior to imaging.

## Description

Superoxide dismutase (SOD) is a key enzyme that converts superoxide into hydrogen peroxide and plays an important role in regulating oxidative stress (Fridovich 1995, Fridovich 1997) .In *C. elegans* , *sod-1* and *sod-5* are predicted to encode zinc–copper superoxide dismutase, and *sod-2* and *sod-3* are predicted to encode manganese superoxide dismutase (Wang, Branicky et al. 2018) . These superoxide dismutase paralogs are present in many tissues, including the nervous system and the intestine (Doonan, McElwee et al. 2008, Horspool and Chang 2017, Horspool and Chang 2018) . Previous work has shown that *sod-5* messenger RNA expression is increased in *sod-1* deletion (Yanase, Onodera et al. 2009) , suggesting that nematode superoxide dismutase paralogs may act to compensate for each other (Doonan, McElwee et al. 2008, Horspool and Chang 2018) . This further complicates the interpretation of experimental results. Data from expression profile analyses and tissue- or cell-specific rescue experiments have provided us clues regarding how these SODs function in overlapping cells (Horspool and Chang 2017, Horspool and Chang 2018) . However, many questions remain unanswered. For example, if all SOD paralogs are present in the same cell, which one will be the predominant SOD in regulating redox homeostasis? Are all four SODs equally important in ameliorating the oxidative stress?

To investigate the role of *C. elegans* superoxide dismutase in the oxidative stress response, we generated a transgene that confers expression of the redox fluorescence reporter rxRFP (Fan, Chen et al. 2015, Fan and Ai 2016) in the intestine. An increase in rxRFP fluorescence intensity indicates an increase in overall oxidative stress. We introduced the rxRFP reporter into *sod-1* ( *tm776* ), *sod-5* ( *tm1146* ), *sod-2* ( *ok1030* ), and *sod-3* ( *tm770* ) deletion backgrounds and measured the fluorescence intensity of rxRFP ( **Fig. 1A** ). We found that rxRFP fluorescence is elevated in *sod-3* deletion. In *sod-1* , *sod-5* , and *sod-2* single deletion backgrounds, the fluorescence intensity of rxRFP remained at the wild-type level. This may be caused by the compensatory effects of other SOD paralogs. To test this, we introduced the rxRFP reporter into double, triple, and quadruple *sod* mutant backgrounds. By introducing *sod-2* into *sod-3* , we found that the rxRFP fluorescence intensity was restored to the wild-type level ( **Fig. 1B** ). Similarly, by introducing the *sod-1* single mutant ( **Fig. 1C** ) and the *sod-1* and *sod-5* double mutant ( **Fig. 1D** ) into *sod-3* , we found that the rxRFP fluorescence intensity was restored to the wild-type level. In contrast, by introducing *sod-1* , *sod-5* , and *sod-2* into *sod-3* , the rxRFP fluorescence intensity remained elevated as in the *sod-3* single mutant ( **Figs. 1E** and **1A** ).

These results suggest that SOD-3 plays a key role in regulating redox homeostasis in the intestine. We hypothesize that SOD-1 , SOD-5 , and SOD-2 may be compensatory for each other. Single and double mutations that consist of *sod-1* , *sod-5* , and *sod-2* may have an increased expression of the non-deleted *sod* loci. As a consequence, it provides a rescue effect for the *sod-3* deletion. Future investigations into the SOD-1 , SOD-5 , and SOD-2 protein levels in various single, double, and triple *sod* mutations may further support our hypothesis or may reveal an alternative interpretation of this observation.

## Methods

**Strains**

*C. elegans* strains were maintained at 20 °C using standard methods (Brenner 1974) . The mutant strains used in this study, including *sod-1* ( *tm776* ), *sod-5* ( *tm1146* ), *sod-2* ( *ok1030* ), and *sod-3* ( *tm770* ), were obtained from the Caenorhabditis Genetics Center and were backcrossed six times with N2 prior to analysis. HCX1537 bosIs1240 [ *sod-1 p* :rxRFP; *ges-1 p* :rxRFP; *unc-122p* ::GFP] were backcrossed before being crossed into *sod* mutant strains. Transgenic strains were isolated by microinjecting plasmids (at 100 μg/mL), together with the co-injection marker *unc-122p::* GFP, into the wild-type. UV integration of the extrachromosomal array was performed following the protocol published by Mitani (Kage-Nakadai, Imae et al. 2014) .

**Molecular cloning**

The rxRFP1.1 cDNA was amplified by PCR using the primers 5’-ATGACTGGTGGACAGCAAATGGGTCGGG-3’ and 5’-AGCCACACTTTTGCACCTTTGGTCAC-3’ and pMito-rxRFP1.1 (addgene #67841) as a template. The mitochondria localization sequence was omitted from the PCR amplification to ensure the expression of rxRFP1.1 in the cytosol. The rxRFP1.1 cDNA was cloned using KpnI/EcoRI sites onto a pUC57 mini vector that contains 3’UTR of *unc-59* . Promoters of *sod-1* and *ges-1* were used to drive the expression of rxRFP1.1 in the intestine.

**Microscopy**

Young adult worms were mounted in M9 with levamisole (10 mM) or with beads onto slides with a 3% agarose pad. The slides were viewed using an AxioImager fluorescence microscope with 20x/0.5 objectives. The fluorescence signals were recorded by a CCD camera in a 16-bit format without saturation.

**Quantification and statistical analysis**

The images were captured and analyzed using MetaMorph imaging software. For all experiments, the significance of differences between conditions was evaluated with GraphPad Prism software. Data sets were analyzed using one-way ANOVA followed by Tukey’s multiple comparison test.

## Reagents

**Table:** 

	SOURCE
Bacterial strains	
Escherichia coli OP50	CGC
Organisms: strains	
*C. elegans* : N2	CGC
C. elegans: GA187 *sod-1* ( *tm776* )	CGC
C. elegans: RB1072 *sod-2* ( *ok1030* )	CGC
C. elegans: GA186 *sod-3* ( *tm770* )	CGC
C. elegans: GA503 *sod-5* ( *tm1146* )	CGC
C. elegans: HCX1537 bosIs1240 [ *sod-1 p* :rxRFP; *ges-1 p* :rxRFP; *unc-122p* ::GFP] 4x backcross	This study
C. elegans: HCX1301 *sod-1* ( *tm776* ); bosIs1240 [ *sod-1 p* :rxRFP; *ges-1 p* :rxRFP; *unc-122p* ::GFP]	This study
C. elegans: HCX1884 *sod-2* ( *ok1030* ); bosIs1240 [ *sod-1 p* :rxRFP; *ges-1 p* :rxRFP; *unc-122p* ::GFP]	This study
C. elegans: HCX1323 *sod-3* ( *tm770* ); bosIs1240 [ *sod-1 p* :rxRFP; *ges-1 p* :rxRFP; *unc-122p* ::GFP]	This study
C. elegans: HCX1880 *sod-5* ( *tm1146 )* ; bosIs1240 [ *sod-1 p* :rxRFP; *ges-1 p* :rxRFP; *unc-122p* ::GFP]	This study
C. elegans: HCX1881 *sod-1* ( *tm776* ); *sod-5* ( *tm1146 )* ; bosIs1240 [ *sod-1 p* :rxRFP; *ges-1 p* :rxRFP; *unc-122p* ::GFP]	This study
C. elegans: HCX1882 *sod-1* ( *tm776* ); *sod-3* ( *tm770* ); bosIs1240 [ *sod-1 p* :rxRFP; *ges-1 p* :rxRFP; *unc-122p* ::GFP]	This study
C. elegans: HCX1883 *sod-2* ( *ok1030* ); *sod-3* ( *tm770* ); bosIs1240 [ *sod-1 p* :rxRFP; *ges-1 p* :rxRFP; *unc-122p* ::GFP]	This study
C. elegans: HCX1896 *sod-1* ( *tm776* ); *sod-5* ( *tm1146* ); *sod-3* ( *tm770* ); bosIs1240 [ *sod-1 p* :rxRFP; *ges-1 p* :rxRFP; *unc-122p* ::GFP]	This study
C. elegans: HCX1887 *sod-1* ( *tm776* ); *sod-5* ( *tm1146* ); *sod-2* ( *ok1030* ); *sod-3* ( *tm770* ); bosIs1240 [ *sod-1 p* :rxRFP; *ges-1 p* :rxRFP; *unc-122p* ::GFP]	This study
Oligonucleotides	
rxRFP cDNA PCR forward primer 5′- ATGACTGGTGGACAGCAAATGGGTCGGG − 3′	IDT
rxRFP cDNA PCR reverse primer 5′- AGCCACACTTTTGCACCTTTGGTCAC − 3’	IDT
Recombinant DNA	
pUC57 simple rxRFP cDNA	This study
Software and algorithms	
GraphPad Prism 9.0	GraphPad Prism Software, Inc
MetaMorph	Molecular Devices
